# Cognitive Functions in Patients after Carotid Artery Revascularization—A Narrative Review

**DOI:** 10.3390/brainsci11101307

**Published:** 2021-10-01

**Authors:** Magdalena Piegza, Gniewko Więckiewicz, Dawid Wierzba, Jacek Piegza

**Affiliations:** 1 Department of Psychiatry, Faculty of Medical Sciences in Zabrze, Medical University of Silesia, 42-612 Tarnowskie Góry, Poland; mpiegza@sum.edu.pl; 2 Independent Public Heath Care Psychiatric Hospital, 44-180 Toszek, Poland; dawidwierzba@gmail.com; 3 Third Department of Cardiology, Faculty of Medical Sciences in Zabrze, Medical University of Silesia, 41-800 Zabrze, Poland; jacek.piegza@gmail.com

**Keywords:** carotid endarterectomy, carotid artery stenting, cognitive functions

## Abstract

Carotid revascularization may lead to improved cognitive function beyond stroke prevention. This article summarizes the conclusions from available studies on the effects of carotid reperfusion procedures on cognitive function. The papers cited used different neuropsychological tests for cognitive assessment, resulting in different methodologies and the results obtained were not always convergent. However, most studies reported an improvement in neurocognitive abilities after both vascular interventions, but a more precise assessment of the specific benefits is still awaited. Clinical determinants to predict the effects of these treatments on cognitive function are still being sought, but results are not yet satisfactory. In view of these studies, carotid stenosis seems to be an independent risk factor for cognitive deterioration, and the main mechanisms responsible are embolism and cerebral hypoperfusion. The aim of this study is to order the knowledge about the effects of carotid artery stenting (CAS) and endarterectomy (CEA) on neurocognitive functions and to verify the usefulness of using these treatments.

## 1. Introduction

According to Global Burden of Disease 2016 Lifetime Risk from Stroke Collaborators, the average lifetime risk of stroke in the United States increased from 22.8% in 1990 to 24.9% in 2016 [1]. In Poland, about 90,000 people suffer a stroke each year; 80% of these cases are ischemic strokes [2] and almost a quarter of them are caused by carotid atherosclerosis [3]. The annual risk of stroke for asymptomatic carotid stenosis narrowing the vessel by 50% is 1%, 80% stenosis carries an annual stroke risk of over 5% [4], while the annual stroke risk for symptomatic stenosis is 13%. Numerous studies show that revascularization of the carotid artery not only protects against stroke, but may also produce an additional beneficial change in some patients: an improvement in cognitive function. The authors of the randomized trial CREST-H propose a new definition of “symptomatic carotid stenosis” that includes cognitive deterioration in its spectrum and involves patients with cognitive impairment that improves after revascularization. These individuals should be treated as a new group of patients who benefit from improvement in carotid artery blood flow [5]. Research published to date suggests cognitive dysfunction in both symptomatic and asymptomatic patients with carotid stenosis. The aim of this paper is to provide a comparative review of the available literature on the effects of carotid artery stenting (CAS) and carotid endarterectomy (CEA) on neurocognitive function in individuals undergoing internal carotid artery (ICA) revascularization. To describe the topic discussed in the title of the article, the authors searched PubMed and Cochrane Library using terms related to the topic. Only English-language articles published after 2005 in peer-reviewed journals were selected. A total of 517 articles were screened (title/abstract) but in the end, 49 articles which were directly linked to the subject of this review were selected, based on the clinical and research experience of the authors.

## 2. The Effects of a Percutaneous Procedure (CAS) and a Surgical Intervention (CEA) on Cognitive Functions

The pathomechanism of cognitive impairment in individuals with carotid atherosclerosis primarily includes: embolization, hypoperfusion, and disseminated demyelinating lesions in the white matter of the brain. Mechanisms leading to neurocognitive deficits in revascularization procedures include: iatrogenic microembolism, atherosclerotic microembolism (so-called silent infarction), thrombosis, cerebral blood flow disturbances and impaired blood supply to the area supplied by a given artery, and hyperperfusion. The overall long-term neurocognitive effect of revascularization is caused by procedure- and patient-related factors. The observed improvement results from both normalization of cerebral blood flow and reduction of emboli.

CAS has been shown to reduce stroke risk in the future, but its effect on cognitive function is uncertain. The vast majority of existing studies report an improvement in neurocognitive abilities after CAS [6,7,8,9,10,11,12,13,14,15,16,17,18,19,20]. One study reports no change [21], while several papers describe a partial or controversial effect [22,23,24]. There are three research papers reporting neurocognitive deterioration after both CAS and CEA [25,26,27]. In the case of CEA, two papers report benefits after vascular surgery [28,29].

One paper showing a partial effect of successful CAS on cognitive function describes a 2017 Chinese study in which carotid artery stenting was performed in asymptomatic patients with at least 70% unilateral stenosis in the ICA. Three months after CAS, improvement was noted in Mini-Mental State Examination (MMSE), verbal memory test, and delayed recall scores. No improvement was noted in digit symbols, MoCA (Montreal Cognitive Assessment), and rapid memory. Blood flow in certain brain regions did improve, but no correlation was found between increased blood flow and cognitive parameters. However, it should be mentioned that the study ultimately included only 16 subjects [22]. A larger number of subjects (N = 579) underwent CAS after cerebral infarction and with carotid stenosis, and then their cognitive functions were measured using MoCA and MMSE at 1 month and 6, 12, 24, and 36 months after CAS. The control group included 552 healthy subjects. Subjects in the CAS group had significantly lower baseline scores on parameters measured by cognitive tests than members of the control group. These parameters improved 6 months after the intervention and remained unchanged or continued to improve after 3 years of follow-up. Age greater than 65 years, low education level, diabetes, and arterial hypertension were found to be independent factors that worsened MoCA parameters 3 years after CAS. The researchers concluded that CAS was associated with significant cognitive improvement in patients after cerebral infarction and with severe carotid stenosis [7]. Grunwald et al. studied 41 asymptomatic, right-handed patients with a burden of arterial hypertension and a control group 24 h before and 3 months after CAS. They performed the following tests: Executive Function Assessment Tests (Trace Recognition Test, Maze Test, Symbol-Figure Test, and Letter Crossing Out Test), Memory Function Tests (Digit Repetition Test, Verbal Memory Test, Visual Memory Test, Delayed Memory Test, and Implicit Learning Test), MMSE, and Beck Depression Inventory (BDI). They also performed cerebral diffusion-weighted magnetic resonance imaging (DW-MRI) to detect microstrokes before CAS and two days after CAS. The results showed significant improvement in cognitive performance (*p* = 0.001) and no changes in memory and verbal functions. These results remained independent of the severity and side of carotid stenosis, as well as the sex and age of the patients. In addition, patients in whom lesions were detected by DWI (34.2%) did not have worse cognitive test scores after CAS than those in whom no such lesions were detected. An improvement in daily activity was also evident [6].

However, Japanese researchers observed differences in cognitive improvement depending on the CAS intervention site (left or right ICA). They showed that intelligence quotient measured by WAIS-III (Wechsler Adult Intelligence Scale III) increased after CAS in patients with severe right stenosis, while verbal intelligence quotient also increased after left ICA intervention [30]. Austrian researchers also found differences in neurocognitive improvement after CAS depending on the side of intervention [17].

The results of numerous studies suggest that cognitive parameters improve several months after revascularization in symptomatic patients with carotid stenosis [10,12]. CAS also improved cognitive function in elderly (62–82 years old) patients with severe stenosis in the ICA, as measured by the MoCA 1 month and 3, 6, and 12 months after surgery. It also improved the patients’ quality of life. The researchers hypothesized that severe carotid stenosis leads to cognitive deterioration and that CAS could improve cognitive parameters and quality of life [13].

A slightly earlier study, published in 2013 and also conducted in the Chinese population, showed a strong correlation between improvement in cerebral blood flow and favorable changes in MMSE and MoCA. CAS improved cognitive function in patients who had carotid stenosis and mild cognitive impairment; the favorable change was closely associated with improvement in cerebral blood flow [14].

A similar dependence of cognitive improvement after carotid stenosis, albeit only in patients with significant cerebral blood flow impairment measured before surgery, was found by Ching-Chang Huang et al. [15]. An earlier work by the same Taiwanese investigators had already found an improvement in cognitive abilities in patients with chronic occlusion of the internal carotid artery after CAS, both in general and in specific aspects (attention and psychomotor function speed) [10]. The recent report from Taiwan focuses on the assessment of functional connectivity (FC) in specific brain structures detected in patients with unilateral stenosis of the ICA and on the evaluation of the effects of FC on cognitive functions. Significant FC abnormalities were associated with worse cognitive parameters, especially memory functions and executive functions, and tended to improve after stent implantation [16]. This is the first project of its kind to investigate compensatory adaptation of the nervous system in patients with carotid stenosis before and after CAS. Lateralization as a compensatory change occurred in patients who underwent CAS in contrast to healthy individuals in the control group. Contralateral functional hypercompensation of the stenosis is how the nervous system copes with the pathology [16].

Studies conducted in recent years have highlighted the impact of cerebral hemodynamic disturbances on cognitive deterioration in advanced carotid atherosclerosis [29,31]. Impaired blood flow due to high-grade carotid stenosis has been shown to be associated with deterioration in cognitive function parameters measured using the MMSE in asymptomatic patients during a three-year follow-up [31]. CAS improves cerebral blood flow and cognitive function in most individuals who had decreased cerebral blood flow and cerebrovascular reserve (CVR) before the procedure [11]. Similarly, patients with severe unilateral ICA stenosis and history of transient ischemic attack (TIA) after CEA showed cognitive impairment specific to the occlusion side before the procedure and significant cognitive improvement and better hemodynamic parameters 6 months after CEA. The investigators hypothesized that cerebral blood supply impairment may be an independent and potentially reversible factor determining cognitive decline in the described patients with severe stenosis [29].

Data from several centers suggest that CAS is associated with a higher incidence of cerebral microembolism compared with CEA [27,32,33]. The total volume of lesions associated with subclinical cerebral microemboli after CAS correlates negatively with changes in cognitive function measured by the RAVLT (Rey Auditory Verbal Learning Test) at short- and long-term follow-up. CAS and low preprocedural perfusion are risk factors for large infarction in the embolization mechanism associated with the procedure. The size of the ischemic lesions significantly determines cognitive performance. The authors suggest that an assessment of neurocognitive status should be performed in every patient with carotid stenosis [33].

Some claim that the neurocognitive state of a post-CAS patient is unpredictable. Various neuroprotection systems are used to minimize the risk of microembolism due to the procedure. Akkaya E. et al. demonstrated an advantage of proximal balloon occlusion system over filter protection. With the former, the researchers observed much fewer new microembolization lesions, and they were not associated with cognitive deterioration [34]. Patients with symptomatic subtotal stenosis of the ICA who underwent CAS with a distal neuroprotection system also showed an increase in global MoCA score as well as attention and delayed memory scores 1 month and 1 year after the procedure compared to the pre-procedure measurement. In the pharmacologically treated group, there was a decrease in these scores at 12 months. In addition, the total score of the MoCA, tracking test, clock test, attention test, and delayed memory test was significantly higher in the CAS group than in the pharmacologically treated group at the corresponding time points [18]. It was found that CAS with a neuroprotection system (flow reversal) is a safe method of carotid revascularization that improves or, in the worst case, does not worsen cognitive function [19]. The risk of microembolization and/or neuroprotection systems are also mentioned by other authors [27,32,33,35,36].

Researchers are looking for clinical indicators to predict the effects of reperfusion procedures on cognitive function. This was the aim of Tani M. et al. who studied eight patients with unilateral stenosis in the ICA before and 6 months after CAS using rs-fMRI (resting-state functional MRI), neuropsychological tests (WAIS-III—Wechsler Adult Intelligence Scale III, WMS-R—Wechsler Memory Scale—Revised) and DMN (default mode network) to assess each patient using an independent parameter determined by rs-fMRI. They measured the correlation between FC and DMN and cognitive changes after CAS. They found no improvement in working memory after CAS, but they showed a negative correlation with FC changes between the DMN and superior frontal gyrus and between the DMN and middle frontal gyrus. Moreover, baseline FC between these brain regions correlated positively with post-procedure improvement in working memory. These results served as a basis for concluding that functional connectivity (FC) between the DMN and dependent region working memory was closely associated with post-procedure working memory improvement; consequently, pre-reperfusion FC assessment might anticipate post-reperfusion working memory improvement in patients treated for unilateral stenosis in the ICA [20].

Some authors try to explain the unclear effects of CAS on neurocognitive functions using the floor and ceiling effect. They believe that the failure to account for this phenomenon in neuropsychological testing limits the interpretability of post-procedural results. The ceiling effect and the floor effect are due to faulty experimental manipulations; this phenomenon is well known in psychology. To avoid these effects, the Austrian investigators decided to exclude patients with extremely high and extremely low test scores from the final analysis. The pre- and post-revascularization scores of the resulting subgroup were compared again. Patients with right-sided and left-sided stenosis were analyzed separately. The tests showed an improvement in verbal and episodic memory and a deterioration in spatial memory. The subgroup of patients in whom the floor and ceiling effect was excluded showed improvement in global cognition (MMSE) and verbal episodic memory (patients with left-sided ICA stenosis) and divided attention (patients with right-sided ICA stenosis) [17]. Authors who assume that only selected functions improve after carotid surgery view their own results with caution and suggest further studies [6,37].

Similar conclusions were reached by Tiemann L. et al. who described both improvement and deterioration in cognitive parameters after CAS in the patients they studied [23]. A comprehensive meta-analysis from 2015, which summarized the results of 16 studies on the effect of CAS alone on cognitive functions, notes a possible improvement in global cognition, memory, attention, and psychomotor speed. No positive effect was found for executive functions, language skills, or functional skills. Nevertheless, CAS was not associated with impairment in any domain of cognitive function [38]. A similar position was taken by other researchers in their original papers [24] as well as Lehrner J. et al. who found no changes after CAS during a six-month follow-up [21].

Other authors performed surgery on symptomatic and asymptomatic patients with ICA stenosis greater than 60%. They found cognitive improvement in MoCA and MMSE after CEA in a group of elderly symptomatic patients with severe stenosis, regardless of which artery was operated. They concluded that CEA does not affect cognitive function and may protect against deterioration [28].

## 3. Comparison of CAS and CEA

As mentioned earlier, a review of the available literature suggests that carotid revascularization procedures improve cognitive performance, with no significant differences depending on the method [37,39,40,41,42,43,44,45]. This view is also supported by Brajesh K. et al. However, when comparing the results obtained by specific individuals in the study conducted by these authors, it is found that the post-CEA group performed better on psychomotor tests, while the post-CAS group performed better on memory and concentration tests. The authors recommend further studies to confirm the specific benefit of carotid artery interventions [39]. It was concluded that the effect of recanalization on cognitive functions is comparable in CAS and CEA by Chinese researchers who studied patients with 70% stenosis using MoCA, MMSE, and P300 (to measure decision-making cognitive functions) and observed an improvement in test scores in the CAS and CEA groups, but found no differences between the two groups [40]. A similar position was taken by Kim et al., who observed a significant improvement in memory and executive functions in patients after vascular interventions [41]. Other researchers recorded an increase in MoCA scores 6 and 12 months after CEA and 12 months after CAS, and no changes in the pharmacologically treated group [42]. In another study, improvement after CAS occurred in episodic, encoded, and working memory, as well as executive functions, while improvement after CEA affected episodic and encoded memory, as well as delayed recall. However, there were no significant differences between the groups, although the CAS group tended to score higher on executive functions [43]. The lack of association between cognitive parameters and ICA stenosis treatment methods has also been observed by other researchers, who concluded that revascularization procedures resulted in cognitive improvement but did not differ in their effect on baseline aspects of cognitive function [37,46]. In the most recent study from 2021, a group of Polish researchers found greater improvement in cognitive function in younger people with poorer baseline cognitive parameters. The only strong predictors of improvement 6 months after surgery were young age and a low MoCA score [47]. Improvement 1 year after the procedure affected visual memory, motor functions, and frontal lobe-related functions and was independent of treatment method. The effect was particularly pronounced in individuals with low baseline cognitive performance [39]. In the CAVATAS and SAPPHIRE studies, no differences were observed between CAS and CEA in terms of neurocognitive functions at long-term assessment [44,45]. Other authors also observed no differences between the effects of the two revascularization methods on neurocognitive processes [46]. A group of only 17 patients (CEA—twelve, CAS—five) with high-grade ICA stenosis but without ischemic lesions before or after revascularization and without significant abnormalities in cognitive tests showed no correlation between blood flow parameters and test scores obtained before the procedures and no significant changes in test scores after revascularization. However, the researchers recorded changes in cerebral blood flow in the hemisphere on the side of the stenosis and found that these changes normalized after reperfusion. The authors themselves state that the result of their study should be considered a success that does not contribute to a clear explanation of the effects of revascularization methods on cognitive performance [47].

Carotid artery vascular procedures reduce the risk of stroke in individuals with high-grade stenosis but are fraught with the possibility of microembolization and memory deterioration after the procedure. Microembolism has been shown to occur more frequently after CAS than after CEA (P 0.005). Fifty-six percent of patients who have procedural embolization show deterioration in verbal memory 1 month later. It was also shown that procedure-related embolization and symptomatic stenosis were independent predictors of a decrease in RAVLT parameters, but only 1 month after the procedure and only in the case of embolization. Six months after the procedure, no association was found between embolization and RAVLT scores. Microembolization associated with the revascularization procedure predicted short-term cognitive deterioration [35]. Cognitive functions were assessed both before and 6 weeks and 6 months after CAS (*n* = 29) and CEA (*n* = 31) in patients with 80% asymptomatic ICA stenosis. Visual and verbal memory as well as attentional functions improved significantly 6 months after the intervention compared to the measurement before the intervention. Cognitive processing speed was better in CAS 6 weeks after surgery, as were executive and motor functions (6 months after surgery). Test scores for attention, memory, and visuospatial skills were similar in both groups (second and third measurements). The authors conclude that revascularization improved memory and attention 6 months later. Compared to CEA, CAS improves cognitive processing speed and executive and motor functions [32].

Some researchers point out the relationship between age and persistence of cognitive deterioration. CAS and age greater than 80 years have been associated with significant long-term cognitive impairment [47]. A slightly different opinion is held by Wasser K., who observed cognitive deterioration in individuals aged 68 years and older and found that it persisted longer in patients after CEA than in patients after CAS [48]. The relationship between the studied parameters and age is also highlighted by other authors [7,26].

Different results were obtained by Watanabe J. et al. who compared the changes in cognitive functions in patients with ICA stenosis of at least 50% both before and 1 year after CEA and CAS with those of patients treated with an antiplatelet agent, using MoCA and MMSE. Interestingly, executive functions measured with the MoCA improved significantly only in patients after CEA, whereas memory measured with the MoCA improved in both the post-CEA and pharmacologically treated groups. In general, both intervention methods improved the MoCA score, but CAS did not cause improvement in any subscale [49].

A similar view on the comparable effect of both procedures on neurocognition has been expressed in previous (2014–2016) reviews [50,51,52]. However, they find both improvement and deterioration after the intervention [50]. This is reflected in a study of a Belgian population in which most patients did not show neurocognitive improvement after CAS or CEA recanalization. Members of all groups showed cognitive deficits before revascularization, but only 10% of subjects showed cognitive improvement 6 months after the procedure and 20% had cognitive deterioration. The revascularization method did not affect the results. The researchers formulated the conclusion that CEA and CAS with neuroprotection systems have a similar effect on cognitive functions [25]. The same opinion is expressed in the work of Altinbas A. et al. who demonstrated cognitive deterioration in symptomatic patients 6 months after both interventions and a lack of dependence on the severity of brain white matter lesions detected by MRI before the interventions. However, patients with more severe, age-related white matter lesions performed worse than patients with less severe lesions, who improved their visual memory scores. Patients participated in the International Carotid Stenting Study (ICSS) [26]. The deterioration of neurocognitive functions after carotid endarterectomy and stenting was described by the same authors 2 years earlier using the same project (ICSS): In that work, they pointed out the lack of statistical differences between methods and a greater number of new ischemic lesions associated with CAS compared to CEA [27].

## 4. Discussion

Carotid atherosclerosis is associated with an increased risk of ischemic stroke and cognitive impairment. Cognitive functions include memory, attention, perception, thinking, language, and communication. Different brain regions play different roles in cognitive processes. Both carotid artery stenting (CAS) and endarterectomy (CEA) prevent future strokes, but their effects on cognitive function remain unclear. No firm conclusions can be drawn from a comparison of the effect of both revascularization methods on cognitive function. Despite several multicenter studies with large patient groups, the results obtained (improvement, worsening, or no effect) and the methods used vary. Chronic cerebral blood supply impairment and silent infarcts are important factors contributing to cognitive deterioration in asymptomatic patients. Procedure-related microembolization is a known complication that occurs more frequently with CAS than with CEA, leading to (usually transient) deterioration in cognitive performance. Improvement in cognitive function after reperfusion procedures can be achieved by reducing embolism and improving cerebral hemodynamics. The above information suggests that minimizing cognitive deterioration in both asymptomatic and symptomatic patients with carotid atherosclerosis may become the new goal of carotid revascularization, in addition to its only goal to date: preventing cerebral strokes. Despite the inconsistent attitude of researchers regarding the effects of revascularization on cognitive function, most of them seem to emphasize the beneficial effects on neurocognition. The authors of the present paper postulate the consideration of an additional indication for revascularization procedures, i.e., cognitive deterioration, possibly in relation to a specifically selected group of patients in whom CAS or CEA may lead to an improvement in neurocognitive functions. Therefore, the search for clinical indicators to predict the impact of the procedures on cognitive functions and the implementation of a mandatory assessment of cognitive function level before CAS and CEA could represent an important new tool for future researchers. Further studies are needed to determine the group of patients who will benefit from revascularization. We also need more sensitive and specific neuropsychological tests that can assign individual functional symptoms to brain regions, as well as modern imaging techniques that are useful for diagnosis.

## 5. Conclusions

- Carotid artery stenosis appears to be an independent risk factor for cognitive decline.
- The main mechanisms responsible are: Embolism and cerebral hypoperfusion.
- Revascularization procedures usually improve cognitive abilities, but CAS is not significantly different from CEA in this regard.
- The question of whether revascularization procedures should be performed to prevent cognitive impairment in individuals with carotid atherosclerosis remains unanswered.
